# Pattern Fidelity of Vertically Aligned GaAs Nanowire Arrays

**DOI:** 10.1002/smll.202506173

**Published:** 2025-10-14

**Authors:** Juliane Koch, Jiajia Qiu, Chris Yannic Bohlemann, David Ostheimer, Huaping Zhao, Peter Kleinschmidt, Yong Lei, Thomas Hannappel

**Affiliations:** ^1^ Fundamentals of Energy Materials Institute of Physics & IMN MacroNano Technische Universität Ilmenau 98693 Ilmenau Germany; ^2^ Fachgebiet Angewandte Nanophysik Institut für Physik & IMN MacroNano Technische Universität Ilmenau 98693 Ilmenau Germany

**Keywords:** AAO, III‐V semiconductor, MOVPE, nanowire, patterning, UTAM

## Abstract

Bottom‐up grown III–V semiconductor nanowire (NW) device components are promising structures for advanced functional electrical and optoelectronic applications, including solar energy conversion devices such as photoelectrochemical cells. The work presents a strategy for preparing highly ordered GaAs NW arrays by combining a low‐cost, non‐lithographic nanostructuring technique with state‐of‐the‐art bottom‐up NW growth via metal–organic vapor phase epitaxy (MOVPE). Regular gold (Au) nanodisk arrays are patterned using an ultrathin anodic alumina membrane mask and serve as catalysts for uniform NW array formation. In contrast to conventional methods such as the expansive, resin‐based nanoimprint lithography, the specific fabrication process of the Au nanodisks prevents undesired substrate imprinting. While previous methods rely on a pre‐anneal nucleation step to preserve pattern fidelity, It is demonstrated that this step alone is insufficient to maintain a well‐ordered array. To overcome this limitation, a pre‐anneal growth step that forms a GaAs pedestal, effectively anchoring the Au particles and stabilizing the array structure, is proposed. A detailed examination of the sequential MOVPE steps facilitates the development of a comprehensive growth model. The refined process paves the way for large‐scale, cost‐efficient production of well‐ordered NW arrays, with potential for integration into solar‐driven water splitting and related applications.

## Graphical abstract

This study presents a fabrication strategy for highly ordered GaAs NW arrays by combining a non‐lithographic nanostructuring technique with metal–organic vapor phase epitaxy. Uniform Au nanodisk arrays, created using anodic aluminum oxide templates, act as catalysts for the subsequent NW growth. A pre‐anneal growth step prior to annealing in the nanowire fabrication is optimized to secure the highly ordered array.

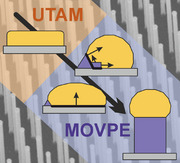

## 1 Introduction

The transition to a sustainable energy economy and the advancement of green electronics are among the most pressing challenges of our time. Nanostructuring of semiconductor light absorbers is a key strategy for enhancing their catalytic activity, reducing material consumption, and improving light exploitation. Nanowires (NWs) offer a promising alternative to planar device architectures due to their unique optical and electronic properties and advantages.^[^ ^1^ ^]^ Their high surface‐to‐volume ratio increases reaction turnover by providing more active sites for photoinduced reactions.^[^ ^2^, ^3^ ^]^ Moreover, their morphology resembles that of light‐trapping optical antennas,^[^ ^4^ ^]^ which enhances both the effective absorption cross‐section and local light concentration.^[^ ^5^, ^6^ ^]^

NWs are being explored as building blocks for a wide range of electronic and photonic applications, including light‐emitting diodes,^[^ ^7^, ^8^, ^9^, ^10^ ^]^ field‐effect transistors,^[^ ^11^, ^12^, ^13^ ^]^ solar cells,^[^ ^14^, ^15^, ^16^ ^]^ and systems for solar water splitting and solar fuel production.^[^ ^3^, ^17^, ^18^ ^]^ A critical factor for commercial viability is the enhancement of light absorption properties, particularly in uniformly ordered NW arrays,^[^ ^19^ ^]^ combined with cost‐effective fabrication and reduced usage of III–V materials. Vertically aligned NW arrays increase light coupling into the absorber and benefit from enhanced light‐trapping. The material choice and geometric parameters of the NW array directly determine its optical and electronic properties – and, hence, the device efficiency – as supported by theoretical modeling.^[^ ^20^ ^]^ Therefore, targeted NW growth is crucial for NW‐based optoelectronic devices that rely on light absorption.

These requirements call for fabrication techniques capable of producing homogeneous, large‐area patterning. Nanoimprint lithography (NIL) has emerged as a high‐throughput method for this purpose,^[^ ^21^ ^]^ but several challenges remain. Photolithographic techniques rely on complex and expensive, specialized equipment, involving time‐consuming and highly precision‐dependent processes. Ultimately, the achievable resolution is fundamentally constrained by the physical diffraction limit of light.^[^ ^22^ ^]^ In addition, volume shrinkage during curing can deform the template and induce compressive stress,^[^ ^23^ ^]^ while capillary forces during imprinting can disturb uniform pattern transfer.^[^ ^24^ ^]^ Even under improved conditions, NIL‐based methods often exhibit non‐uniformity in Au catalyst arrays.^[^ ^25^ ^]^

A promising alternative involves the use of anodic aluminum oxide (AAO) membranes, such as ultrathin alumina membranes (UTAM), which allow for scalable and tunable surface nanopatterning.^[^ ^26^ ^]^ Owing to their low equipment requirements, cost‐efficient fabrication, and rapid processing, UTAMs represent highly versatile nanotemplates for the reliable production of wafer‐scale nanostructure patterning exhibiting outstanding structural uniformity and periodicity.^[^ ^26^, ^27^, ^28^, ^29^ ^]^ Here, we employ a novel fabrication strategy for vertically aligned III–V semiconductor NW arrays by combining UTAM‐based catalyst patterning with bottom‐up vapor‐liquid‐solid (VLS)^[^ ^30^ ^]^ NW growth in a metal–organic vapor phase epitaxy (MOVPE) system. The Au nanodisks, defined by the UTAM mask, act as site‐selective catalysts for uniform NW formation.

To preserve the array arrangement during MOVPE, an additional process step is introduced: a pre‐anneal growth step that stabilizes the position of the Au particles. This builds on nucleation concepts originally developed for NIL‐defined arrays,^[^ ^25^ ^]^ but must be adapted to account for differences in the substrate imprinting and Au volume in UTAM‐based structures. Through a detailed examination of the individual MOVPE sub‐steps, we derive a comprehensive growth model. While the procedure is illustrated using the GaAs material system, it is reasonable to assume that the methods presented are also applicable to other III–V semiconductor materials. Metal particle patterning can be performed using various metals,^[^ ^27^, ^31^, ^32^ ^]^ and VLS‐based NW growth has been successfully employed for a wide range of NW materials.^[^ ^33^, ^34^ ^]^ This approach enables precise control over NW geometry and array order – crucial for tailoring optical and electrical properties to specific applications.

## 2 Results and Discussion

**Figure 1:**
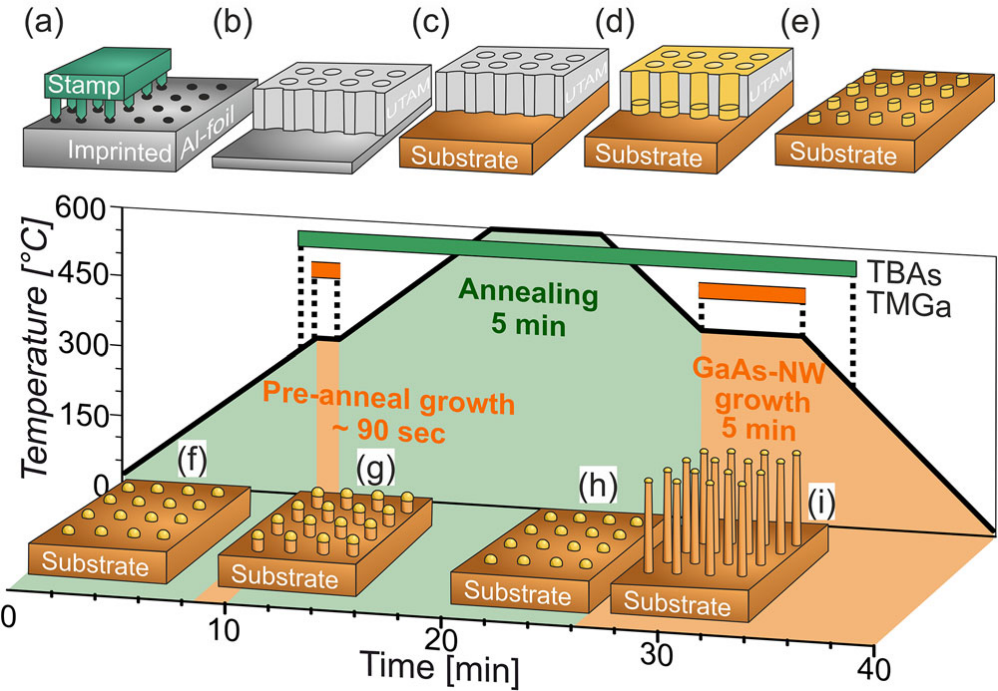
Combination of Au structuring (UTAM) and Au catalyst‐assisted NW array growth (MOVPE). a) Embossing electropolished Al foil. b) Anodization. c) Back‐side Al removal, pore expansion and transfer to the III–V substrate. d) Au deposition (PVD). e) UTAM layer removal, retention of the Au disks. f) Heating up and contraction of the Au particles. g) Pre‐anneal growth step with NW pedestal growth. h) Annealing and reabsorption of the III–V pedestal. i) NW growth.

We fabricated UTAM masks with typical dimensions^[^ ^26^ ^]^ and utilized them for the deposition of regular arrays of Au disks, which can be used in the next step as a catalyst for the NW growth in a horizontal, low‐pressure MOVPE reactor. The UTAM fabrication process comprises several steps,^[^ ^35^ ^]^ which are shown in **Figure** **1**, including an electropolished aluminum (Al) foil imprinting, followed by anodization, pore opening, and transfer to the growth substrate. After depositing Au using physical vapor deposition (PVD), the UTAM layer is removed, leaving Au disks on the substrate. Additional information on preparing the UTAM mask can be found in Section 4 (Experimental Section).

These Au disks serve as catalysts in the subsequent MOVPE process. However, the displacement and coalescence of Au particles during annealing prior to actual NW growth is a major obstacle to the fabrication of dense NW arrays. Otnes et al.^[^ ^25^ ^]^ proposed strategies to improve the preservation of the order of the particles. In contrast to their work, which employed NIL to define the catalyst disks, we choose UTAM as a structuring method, and therefore some differences in the preservation of the ordering have to be expected. Many publications using NIL,^[^ ^36^, ^37^, ^38^, ^39^ ^]^ electron beam lithography,^[^ ^40^, ^41^ ^]^ or displacement Talbot lithography^[^ ^42^ ^]^ follow the strategy of fixing the catalyst by altering the growth process prior to annealing by a pre‐anneal nucleation. We have developed a modified strategy, which we will refer to as the pre‐anneal growth step. At this step, similar to conventional NW growth but at a reduced temperature of 320 °C, a supersaturation of the Au disks occurs due to the introduction of group III and V precursors into the MOVPE reactor. By extending the pre‐anneal growth step up to 120 s, a small NW pedestal is grown. The adjusted MOVPE process for the UTAM‐structured Au disks is illustrated in Figure 1(f–h), with the process steps of pre‐anneal growth, annealing, and NW growth aimed at fabricating free‐standing NWs. More details about the MOVPE sample preparation are given in Section 4 (Experimental Section).

**Figure 2:**
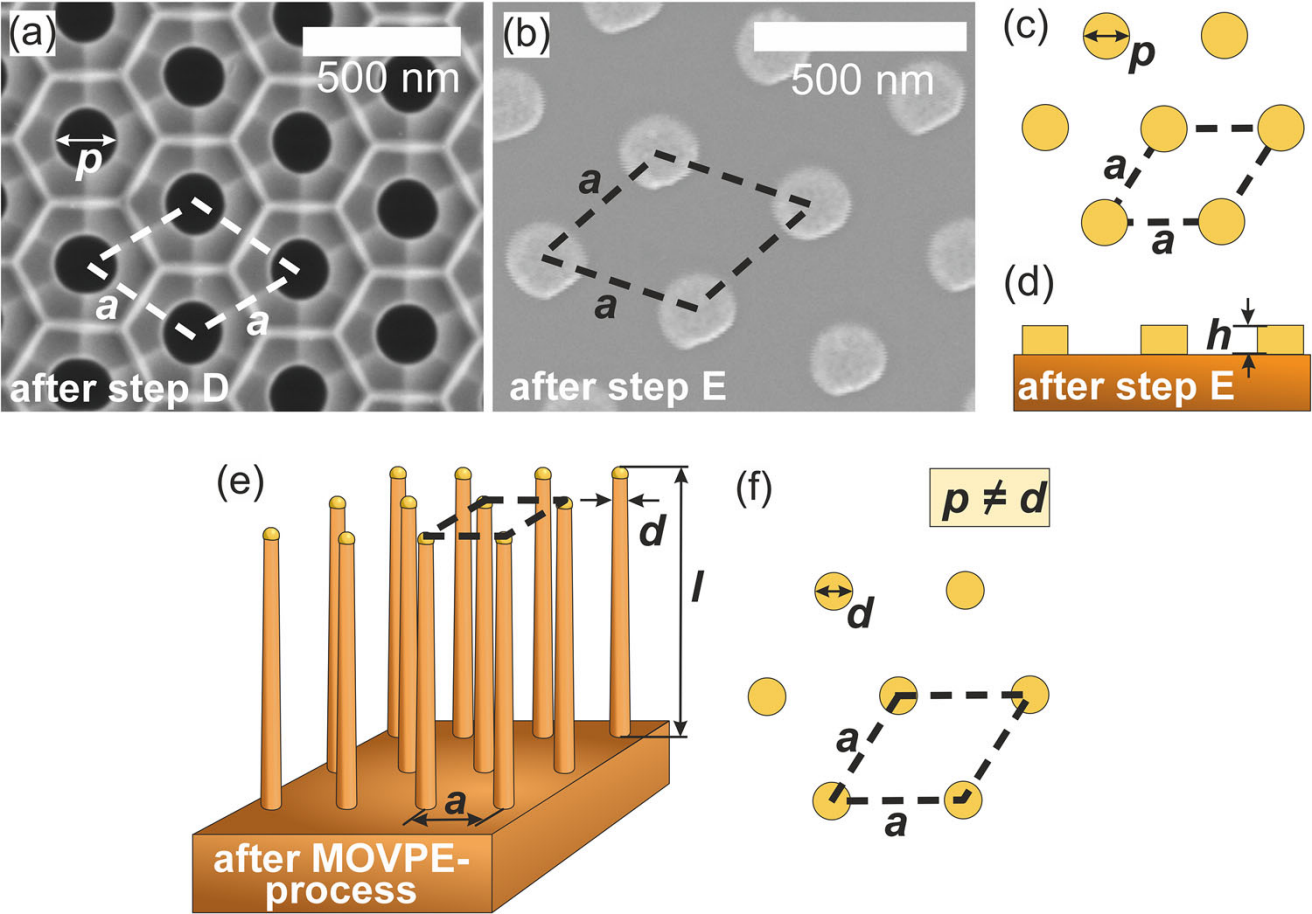
Illustration of the geometry parameters using SEM images in a), b), and sketches in c–f). a) SEM image taken after step D of Figure 1, showing the pore spacing *a* (400 nm for all samples) and the diameter of the pore opening *p*. b) SEM image after UTAM removal, revealing remaining Au disks with the same diameter as the pore size *p* of 200 nm. c) Sketch of b) for clarity. d) Side view sketch of b), c). The height of the Au disks can be obtained by AFM measurements (see Figure S1, Supporting Information). e) Illustration of a NW array after the MOVPE process in a tilted side view. f) In‐plane view sketch of e). In the ideal case, the NW spacing remains unaffected by the MOVPE process. The length *l* of the NW is determined by the growth duration. The geometry of the Au particles changes due to heat‐induced surface energy minimization. While the diameter of the Au particle or NW does not correspond to that of the Au disk, while the volume of the Au remains unchanged.

Now, this approach is used for the fabrication of the samples employed in this work. The resulting samples can be characterized by the following standardized parameters. These parameters are illustrated in **Figure** **2** through scanning electron microscope (SEM) images and descriptive sketches. In Figure 2(a), the SEM images visualize the pore spacing *a* and the pore opening *p* of the UTAM. All samples utilize a pore spacing *a* of 400 nm. The final diameter *d* of the NWs grown in the MOVPE process is directly determined by the size of the Au particles after annealing. In our approach, two parameters are varied to control the initial volume of Au deposited by PVD on the substrate. The pore opening *p* of the UTAM mask defines the lateral size of the Au disks, and the thickness of the evaporated Au layer determines the vertical Au disk height *h*. These two parameters define the initial Au volume, which determines the volume of the final Au particle geometry after annealing. During heating in the MOVPE process, surface energy minimization is expected to cause the formation of near‐spherical Au particles with numerous facets, indicating a shape transformation of the Au particle. This implies that, although the Au volume of the Au disk corresponds to that of the Au particle, the diameter and height change. Consequently, the pore opening *p* is not equal to the diameter *d* of the particle after the MOVPE process. Since the diameter of the Au particles after the annealing step determines the diameter *d* of the NWs, the controlled variation of the disk height and the pore size allows a systematic tuning of the NW geometry.

In the initial experiments, the pore opening *p* was set to 200 nm, whereas in the final experiment, a smaller pore opening *p* of 135 nm was used. An SEM image after removal of the UTAM with remaining Au disks is depicted in Figure 2(b). In addition to the pore size *p*, several Au disk heights *h* are used, as illustrated in Figure 2(d). These include the values of *h* ≈25 nm as well as *h* ≈34 nm.

By fixing the Au particles in their grid positions, the distance *a* should ideally be kept constant during the MOVPE process. The length of the NW is determined by the process time, temperature, and the amount of the group III and V precursors introduced during the MOVPE processes.

It should be emphasized that the results of Au particle arrays fabricated with UTAM differ significantly from the samples fabricated with NIL, where the array arrangement is almost 100% preserved after NW growth,^[^ ^25^ ^]^ which is generally not the case for UTAM prepared samples. It can be assumed that the NIL‐prepared substrates were pre‐structured by the imprinting stamp during the structuring process, resulting in a depression into which the catalysts were deposited. This constitutes a barrier to particle diffusion along the substrate surface, enabling an ordered arrangement of the NWs to be achieved, even without a pre‐anneal growth step. In contrast, the Al foil in the UTAM‐prepared samples is structured prior to being transferred to the III–V substrate. This ensures that the GaAs substrate remains unaffected by the imprinting stamp, so there are no depressions, and this additional diffusion barrier is not introduced. Based on this, diffusion of the Au particles along the unaltered substrate surface is likely to occur without a pre‐anneal growth step.

**Figure 3:**
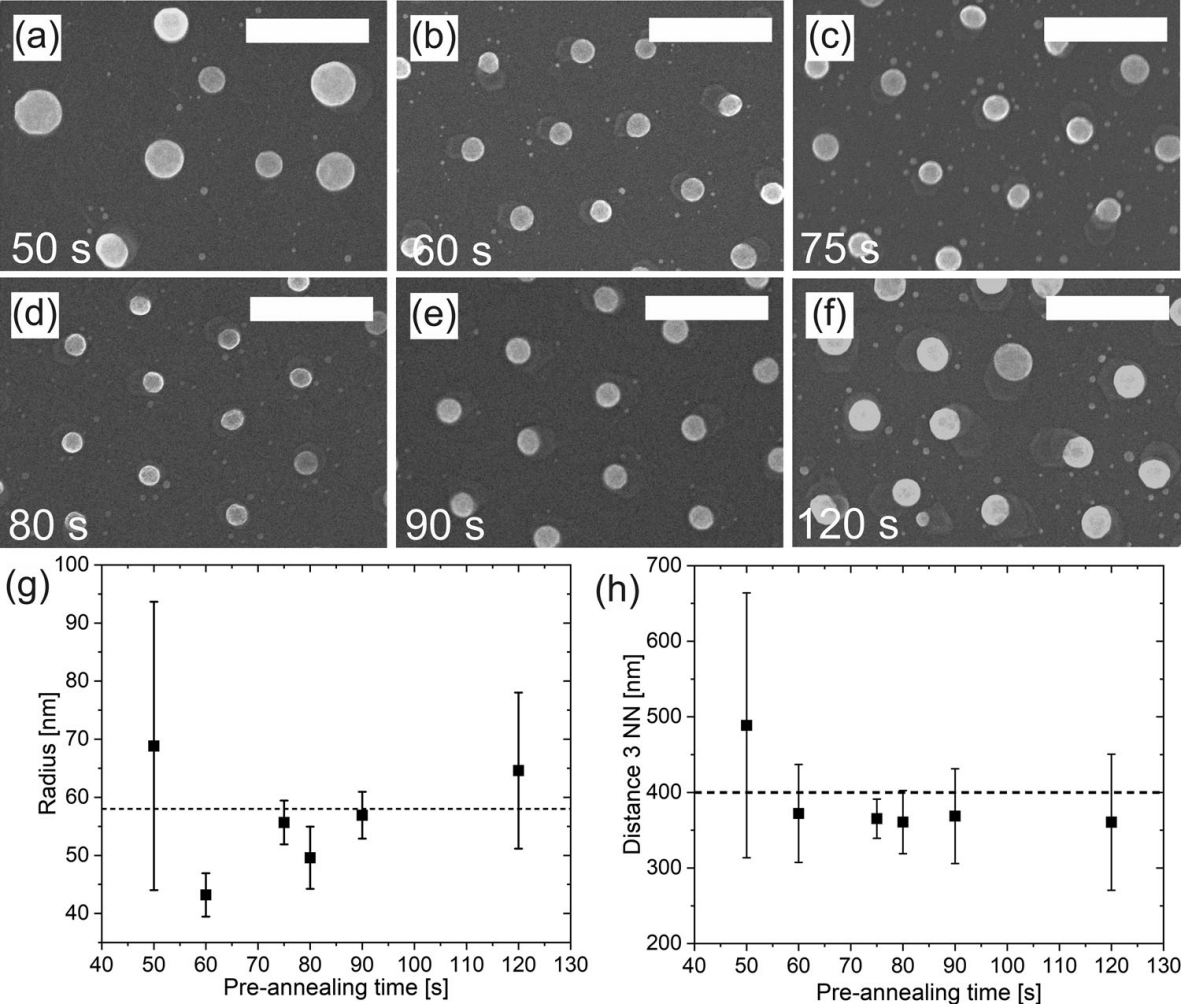
Influence of the pre‐anneal growth time on the particle movement at samples with *h* ≈25 nm. a–e) SEM images of samples with varying times from 50 to 120 s. f) Measured average radii with error bar within each sample a–e). g) Measured distance of the next three neighbors for each sample a–e). Scale bars are 500 nm.

To clarify the influence of this additional pre‐anneal growth step on suppression of lateral diffusion and particle anchoring, the pre‐anneal growth duration was varied in **Figure** **3** between 50 s in (a) and 120 s in (f), followed by an annealing step at 600 °C for 5 min, to ensure the removal of the oxide layer, which is crucial for successful vertical NW growth.^[^ ^43^ ^]^ In this sample set, *h* and *p* vary by 5%, resulting in different Au particle volumes within this sample set. The areas on the sample that show the largest possible particle volumes in a grid arrangement are selected during the examination.

If the pre‐anneal growth time is kept short (e.g., 50 s, Figure 3(a)), the Au disk grid arrangement is not maintained. The particles diffuse along the substrate surface after the pre‐anneal growth step, especially during the annealing, and agglomerate to form larger particles. While at 60 s, Figure 3(b), a clear structure can be seen, which is repeatedly interrupted by clearly displaced particles, within the range from 75 to 90 s, Figure 3(c–e), a higher order of the particles can be seen. If larger pre‐anneal growth times are considered, a critical threshold appears to have been exceeded. In the SEM image for 120 s (Figure 3(f)) a greater disorder can clearly be seen. Therefore, the long‐range order of the Au disk grid shows a certain dependence on the pre‐anneal growth procedure. Clearly, the fine adjustment of the pre‐anneal growth time is crucial for preserving of the Au particle arrangement.

To enable a quantitative comparison between the samples, the relative occurrence of arrangement errors is shown by measuring the Au particle radii *r* in Figure 3(g) on the one hand and the distance to the nearest three neighbors (abbreviated: 3 NN) in (h) on the other hand. For a better statistical evaluation by a custom Python script, larger substrate surfaces with more than 150 particles were used to determine more precise characteristic values than in the SEM images shown in Figure 3(a–f).

The expected values for the distance to the 3 NN were determined when using a pore spacing *a* of 400 nm. Assuming *p* > *h*, *p* led to variations in *d*. Initially, a flat cylinder was assumed for the Au deposition, but AFM images (see Figure S1, Supporting Information) suggested that a spherical segment was a more accurate model. The values of *h* and *p* together determined the volume of the Au particle and the subsequent diameter of the NW. Therefore, the volume of the Au particle after the MOVPE process can be estimated by $V_{half-sphere}=\frac{1}{12}\pi \;{\left(d\right)}^{3}=\frac{2}{3}\pi {\left(r\right)}^{3}$, which should be equal to the volume of the original Au particle after the PVD deposition process $V_{sphere\;segment}=\frac{1}{8}h*\pi {\left(p\right)}^{2}+\frac{\pi }{6}{\left(h\right)}^{3}$. Thus, the subsequent radius *r* of the Au particle after the MOVPE process can be estimated to be $r=\sqrt[{3}]{\frac{3}{2}*\left(\frac{1}{8}h*{\left(p\right)}^{2}+\frac{1}{6}{\left(h\right)}^{3}\right)}=57.6\;nm$.

With the estimated comparative value, the radii of the particles from Figure 3 can now be evaluated more precisely. Shorter times (e.g., 50 s) result in insufficient particle anchoring, leading to the diffusion and agglomeration of Au particles, causing significant variations in individual radii, and complete disorganization of the grid arrangement, as indicated by the 3 NN distance analysis. This lack of long‐range order highlights the inadequacy of short pre‐anneal growth periods.

At a pre‐anneal growth time of 60 s, particles with a radius of ≈43 nm are partially stabilized, although their radius and volume remain below the expected values (*r* ∼58 nm), likely due to inhomogeneities in the pore sizes of the UTAM mask.

When the pre‐anneal growth time is increased to 75 s, the Au particles begin to stabilize within the target radius range. Extending the time to 90 s results in more consistent particle sizes and a well‐maintained grid arrangement, with minimal variation in the 3 NN spacings, reflecting better long‐range order. Between 75 and 90 s there is a range where many particles have the expected radius and show only a small scatter around the mean value. The 3 NN distance is also in the range of the expected value of 400 nm. It should be noted that with a longer pre‐anneal growth time, both small and larger particles are addressed, and therefore, small differences in Au volume are less significant for the particle distance. This leads to more precise and homogeneous results at 90 s than at shorter times.

However, an excessive pre‐anneal growth time of 120 s leads to outgrowth and the displacement of Au particles from their original array sites occours, causing significant spatial rearrangement and deviations from the expected particle size. Furthermore, the pre‐anneal growth time is long enough that very large Au particles, larger than the expected value of 58 nm, can be addressed. It should be noted that larger‐than‐expected Au particle volumes can also occur due to variations in initial particle volumes.

The results clearly show that the Au particle radius strongly depends on the pre‐anneal growth time. Therefore, the pre‐anneal growth time is crucial for maintaining an Au particle grid arrangement and must be adjusted for a given volume of Au particles.

**Figure 4:**
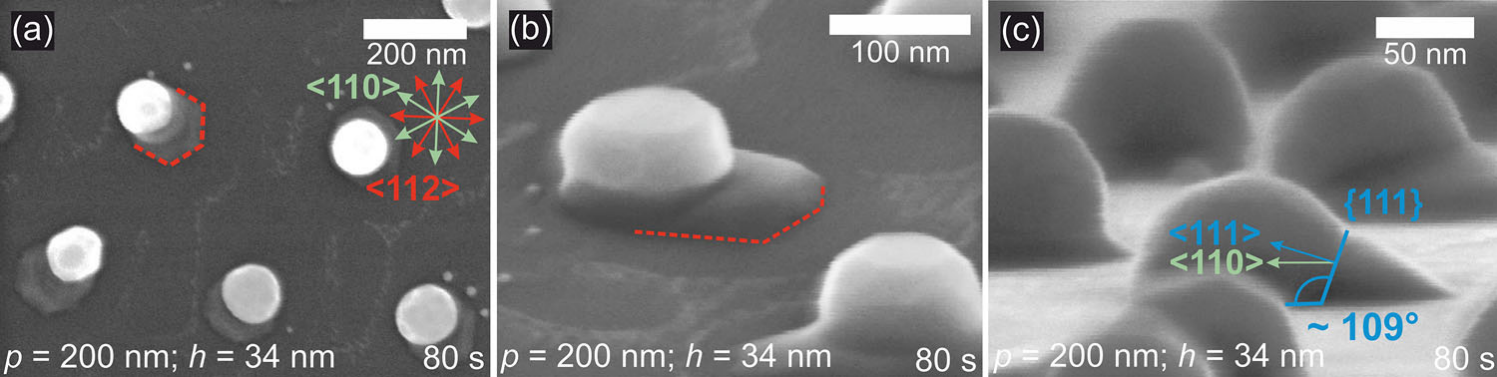
Illustration of the crystallographic directions at the pedestal. SEM images of a sample with *h*=34 nm and a pre‐anneal growth time of 80 s at 320 °C, followed by annealing at 600 °C for 5 min. a) Top view. b) Another particle for comparison at a 70° tilt. c) Determination of the contact angle between the Au particle and the pedestal. Sample tilted by 85°.

In addition to analyzing the long‐range order of the Au disk lattice, the SEM images in Figure 3 also show that pedestal structures have formed around the Au particles. Extended pedestal areas can be seen as planar‐grown, wedge‐shaped GaAs structures. Moreover, all particles appear to exhibit a preferred direction during pedestal growth, which becomes more pronounced with longer pre‐anneal growth times. A detailed examination of the base area shows that it has an angular structure. This can be seen very clearly in **Figure** **4**, where a sample with an Au disk height *h* of 34 nm, a pre‐anneal growth time of 80 s at 320 °C, and an annealing time of 5 min at 600 °C was used. This suggests that the edges of the pedestal adopt the same crystallographic directions as the surface of the substrate, as can be seen in the SEM image of the sample edge (see Figure S2, Supporting Information). The (111) surface has a hexagonal structure with six symmetrically equivalent <110> and six symmetrically equivalent <112> directions, as shown in Figure 4(a), top right). The base region of the particle in Figure 4(a,b) exhibits edges aligned with the {112} crystal planes. The Au particle moves forward along the <110> direction due to material deposition at the Au‐pedestal interface. While the wedge structures vary in size, they generally maintain a consistent contact angle with the Au particle, likely corresponding to a {111} interface with low interfacial energy.^[^ ^44^ ^]^ The observed contact angle of 109° in Figure 4(c) closely aligns with the theoretical {111} angle of 109.5°,^[^ ^45^ ^]^ indicating an {111}A growth facet and a <110> growth direction. Comparison of the base edges across samples reveals a {111}B growth facet in a few particles with a theoretical angle of 70.5°.^[^ ^45^ ^]^ Furthermore, no preferred growth direction between <110> and <112> is observed, probably due to GaAs homoepitaxy, in contrast to heteroepitaxial systems, where <112> dominates to reduce internal stress.

**Figure 5:**
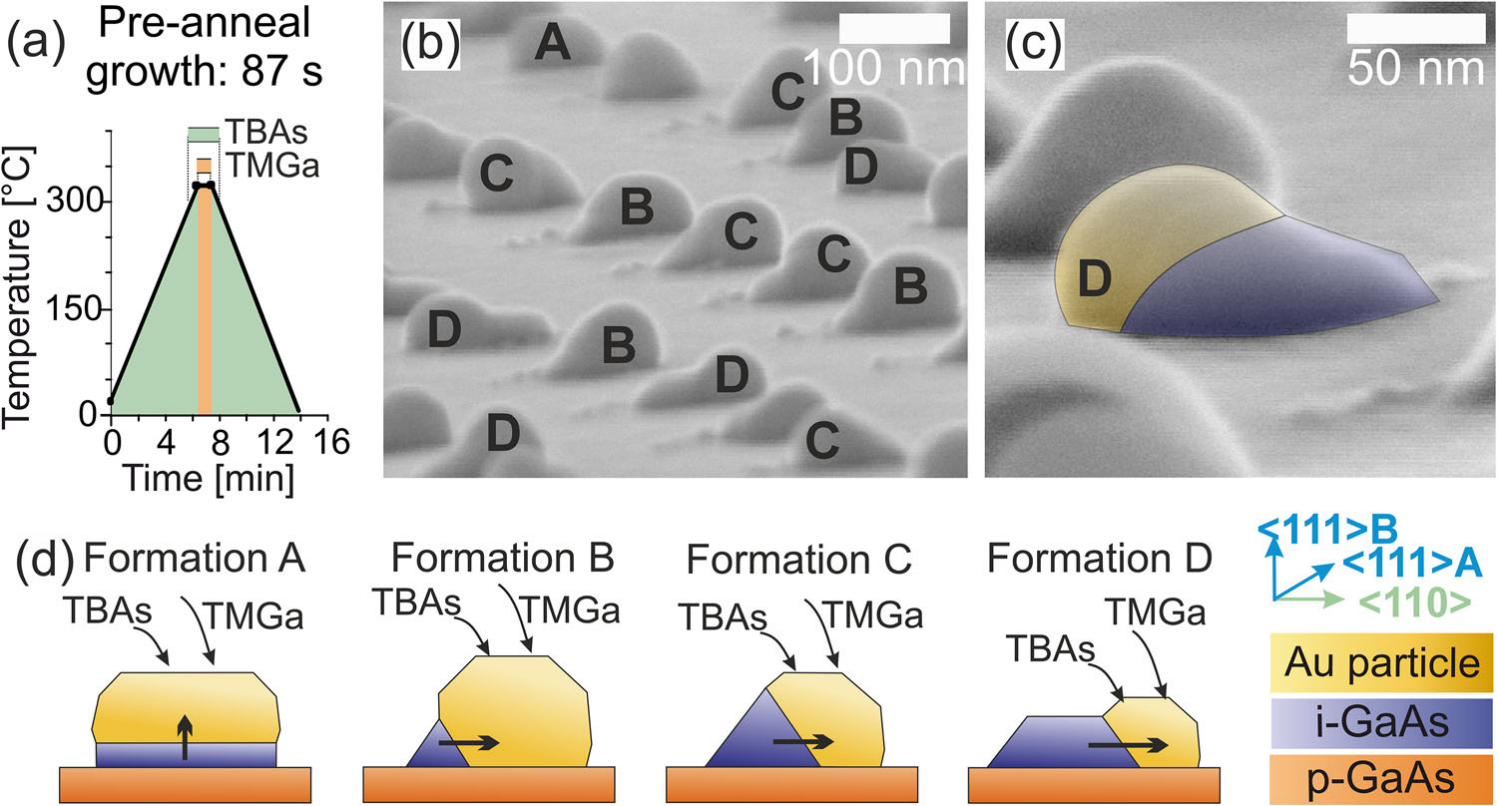
Analysis of the pre‐anneal growth step of 87 s at 320 °C of a sample with *h*=34 nm. a) Schematic representation of the temperature‐time profile. b) SEM image under 85° tilt with formations A to D marked. c) SEM detail of formation D. Image taken under 85° tilt. d) Illustration of formations A to D. Arrows indicate the direction of growth.

To clarify the origin of these differences, SEM images were taken of a sample after pre‐anneal growth, without an annealing step, as shown in **Figure** **5**. A sample with a pre‐anneal growth time of 87 s was analyzed, dividing the base areas formed by vapor‐solid‐solid (VSS) growth into four formations (A‐D) in Figure 5(b). Formation A occurs relatively rarely and stands out, resembling the original particle shape after PVD, characterized by a large diameter and low height. These particles exhibit vertical pedestal growth in the <111> B direction with minimal contraction, maintaining a single {111} facet on top, as illustrated in (d). Crystallization of GaAs from the Au particle takes place over the entire contact surface of the Au particle with the GaAs substrate. In contrast, formations B, C, and D display more pronounced lateral GaAs pedestal growth, with the Au particles becoming increasingly spherical and the Au particles leaving their original location due to VSS growth, thus exhibiting some mobility despite the relatively low process temperatures.

Formation B features a small, wedge‐shaped base with minimal particle displacement, while formation C shows greater lateral growth and displacement with a reduced Au particle volume, as illustrated in Figure 5(d). Formation D, with the smallest Au volume, exhibits the most significant displacement and an elongated base area surface in the <110> direction. The observations indicate that the initial particle volume determines the growth type. In formations B, C, and D, the Au particles contract during the pre‐anneal growth step, forming spherical structures with facets, while the particles in formation A retain their initial shape. This suggests that gallium (Ga) lowers the melting point of Au and forms a Ga–Au alloy, leading to a phase transition of Ga, but their different volumes result in different Ga‐Au ratios. Figure 5(b) shows inhomogeneities in particle diameter and volume within a sample. The volume of the Au particle also influences the pedestal structure. Larger particles reach supersaturation with III and V components later than smaller ones, resulting in a smaller base area. Smaller particle volumes lead to earlier pedestal growth, and in very small volumes, as seen in formation D, rapid supersaturation causes significant planar GaAs pedestal growth. Since the precursor supply time is constant, this results in various pedestal structures with different growth rates.

Another factor may be the native oxide layer beneath the Au particle, which has not yet been considered. Complete removal of this oxide typically requires temperatures above 500 °C, whereas the 320 °C used for pre‐anneal growth is unlikely to be sufficient for complete removal. This incomplete oxide removal could also affect the pedestal structures in addition to the factors mentioned above.

**Figure 6:**
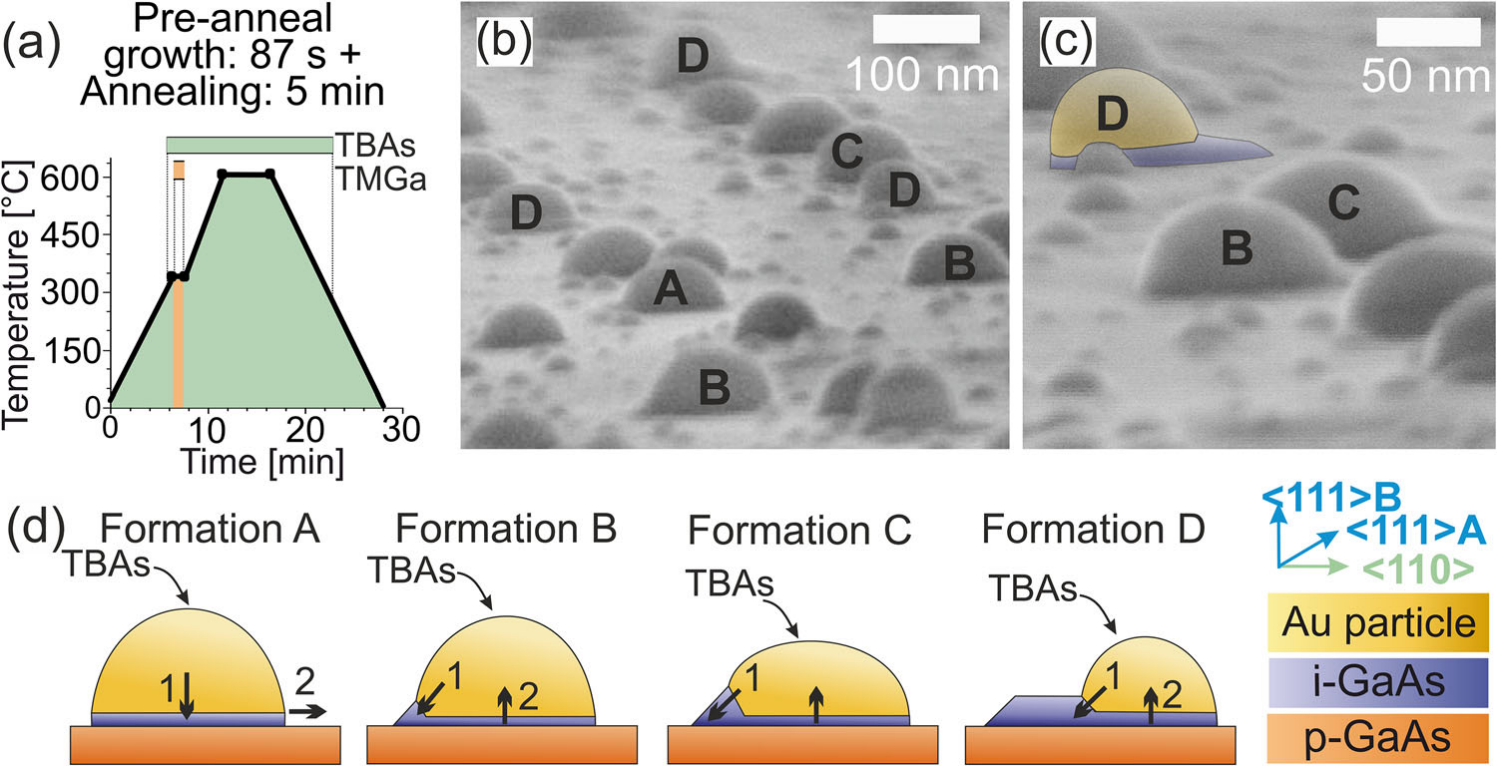
Analysis of the 5 min annealing step at 600 °C after the pre‐anneal growth step with 87 s at 320 °C of a sample with *h*=34 nm. a) Schematic representation of the temperature‐time profile. b) SEM image under 85° tilt with marked formations A–D. c) SEM detailed image of formation D. Image under 85° tilt. d) Illustration of formations A–D. Arrows indicate the growth direction.

In addition to the previous analysis of the pre‐anneal growth step, the impact of annealing on different formations is examined by adding a 5 min annealing step at 600 °C after the 87 s pre‐anneal growth at 320 °C, as shown in the temperature‐time profile in **Figure** **6**(a). At this higher temperature, the Au particle is likely to enter a liquid phase due to the reduced melting point caused by the addition of Ga, and Ga may diffuse from the GaAs substrate into the particle. SEM images at a high tilt (85°) in (b) and (c) reveal increased disorder and a rounder shape of the Au particles, confirming that VSS, not VLS, growth occurred during the pre‐anneal growth step.

The initial formations evolve further after annealing. In formation A, the SEM image does not show a distinct base area, as the Au particle absorbs the base (almost) completely and makes full contact with the GaAs substrate. This allows the particle to diffuse across the surface and lose its original position. In formations B and C, the Au particle significantly decreases in height and increases in width, with a more pronounced effect in C. The lateral GaAs pedestal is partially reabsorbed, resulting in a significant reduction in the volume of the base area.

However, the annealing time is insufficient to completely absorb the base area. Given the same annealing time for all formations, the remaining base areas in B and C differ, with formation C retaining a larger base area due to its initially larger size. In formation D, the Au particle reabsorbs the lateral pedestal during annealing, but its volume limits the uptake of the group III and V components, making the particles more spherical than in B and C.

Overall, the annealing time limits the absorption of the base material, while the particle volume restricts further uptake due to the saturation capacity of the group III and V components. Cooling to room temperature after annealing for SEM imaging likely causes the Au particle to transition from liquid to solid, leading to GaAs recrystallization and the formation of a new pedestal region at the particle‐substrate interface. This can be seen in a direct comparison for formation D in Figures 5(c) and 6(c). This process is also to be expected in a cooling step of a conventional NW growth process when the sample is cooled from 600 °C to the growth temperature of ≈430 °C for subsequent NW growth.

These observations of the pedestal formations after cooling are directly related to the different base structure formations and their evolution during the annealing. In this context, formations B and C are particularly desirable for the production of an NW array arrangement, while formation A is unsuitable due to the rapid absorption of the III–V base and diffusion along the substrate surface. Formation D is also unfavorable for the production of an ordered structure due to the strong planar base growth.

**Figure 7:**
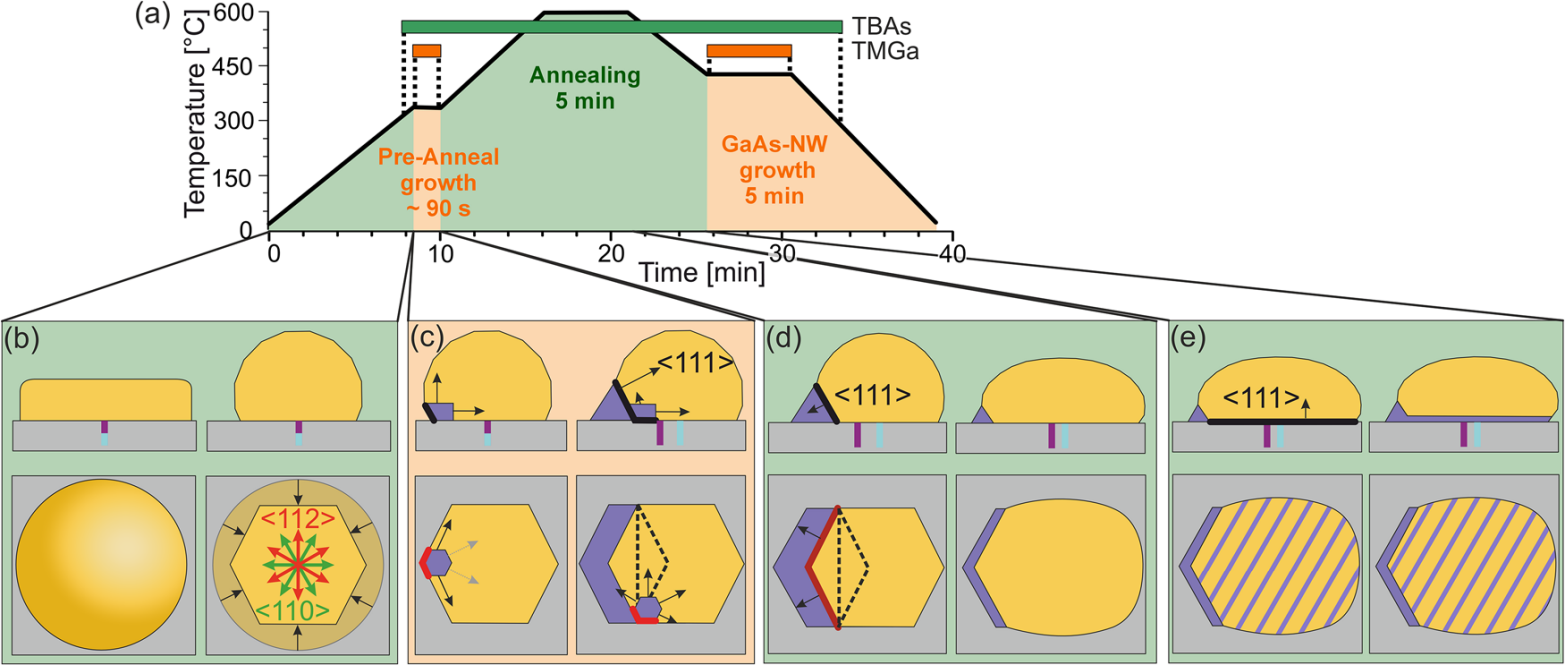
Schematic representation of the growth and absorption processes that take place during the sub‐steps of the MOVPE process. a) Temperature‐time profile. b) Starting point after the PVD process and initial heating to 320 °C. c) Growth processes during growth at 320 °C for ≈90 s. d) Absorption processes to liquify the Au particle during heating to 600 °C and subsequent annealing for 5 min. e) Cooling to the NW growth temperature of 430 °C. The upper diagrams in b–e) show the particles in side view, whereas the lower diagrams show the top view. The black arrows show directions for contraction in b), growth in c), absorption in d), and re‐growth in e). The multi‐phase boundaries are highlighted with red lines. The black dashed lines in c) and d) are intended to illustrate the possible growth facets.

Based on the previous findings, a uniform growth model for the base area during the different MOVPE steps is developed, highlighting the atomic processes involved. The temperature‐time profile of the entire MOVPE process is illustrated in **Figure** **7**(a). All key stages of pedestal growth are depicted in Figure 7, with the initial and current positions of the Au particles shown to visualize deviations from their grid arrangement. This model applies primarily to formation B but also extends to formations C and D.

At the start of the MOVPE process, the Au particle is solid, and disk‐shaped, and its size is defined by the UTAM mask and the PVD process, as depicted in Figure 7(b). During heating to 320 °C, the particle contracts to a near‐spherical shape to minimize the surface energy, though it remains faceted. The interface of the particle with the substrate is likely to align with hexagonal facets, such as {112} or {110}.

As the pre‐anneal growth step begins at 320 °C, Ga accumulates in the Au particle until supersaturation triggers condensation into the solid phase, driven by the chemical potential difference between the phases. Growth nuclei form within the particle, but only lead to growth if energetically favorable. Pedestal growth occurs when the nucleation rate *J_i_* or probability *p_i_* of a 2D nucleus is higher due to a lower Gibbs energy difference $\Delta G_{i}^{*}$, which acts as a barrier to stable nucleus formation. During epitaxial crystallization of the growth component, the nucleation position is most likely to be at the corners of the hexagonal particle base. At this position, marked by the red line in Figure 7(c), the three‐phase boundary between the gas phase, substrate, and particle minimizes the interfacial energy of the Au particle and promotes nucleation compared to positions inside the particle. The higher arsenic (As) concentration in these areas further increases the nucleation probability at the Au particle edges. Unlike Ga, As can only alloy with Au in small amounts (1%–3%) and diffuses mainly to the outer regions of the particle. Assuming that homoepitaxy occurs, the new nucleus facet matches the previous interface, marked by a black line in Figure 7(c). After the first nucleus forms, the nucleation probability elsewhere decreases as the growth component concentration drops, leading to VSS growth at the initial nucleus site. Step‐flow growth, typical of VLS‐based NW growth, might be expected, where growth species attach with minimal energy barriers and expand the base area uniformly. However, this only applies to particle formation A. For formations B to D, a wedge structure forms early, hindering uniform pedestal formation, as seen in Figure 5(b).

To better understand this behavior, it is important to consider the factors that may contribute to the formation of the wedge‐shaped structure. A slight substrate misorientation of ± 0.5° can promote the development of {111} growth facets. Once this facet is established, any change in growth direction is subject to an energetic barrier. During pre‐anneal growth at 320 °C, the Au particle remains solid, as shown by the different particle shapes before and after annealing in Figures 5 and 6, indicating VSS growth. At this temperature, the precursors are not fully decomposed, leading to a lower growth rate than conventional NW growth at 430 °C. Changes in crystallographic structure (ZB to WZ) can also introduce growth defects. Lattice strains are reduced by a wedge‐shaped growth front that lowers the interfacial energy. The most energetically favorable growth plane in this case is the {111} plane. Once the {111} front forms, growth proceeds steadily in the <112> direction.

This behavior is consistent with vertical NW growth at 430 °C, where no planar growth occurs due to thermal energy release of lattice stresses, making vertical growth energetically favorable. Another factor could be the favorable three‐phase boundary, where a new nucleus forms below the Au particle, promoting island growth at the edges of the particle due to the lower As concentration in the center. Figure 4(c) shows a {111}A growth front angle of 109.5°, while the samples as a whole also exhibit a B polarity angle of 70.5°, likely influenced by initial conditions such as substrate misorientation and particle shape. Once the initial {111}A growth front is established, growth continues accordingly in all cases. The highest probability of the next nucleus position to minimize the interfacial energy corresponds to a position at the four‐phase boundary of the substrate, pedestal, Au particle, and gas phase. Consequently, the pedestal structure grows in the <112> or <110> direction parallel to the substrate surface.

Based only on the SEM measurements, we cannot rule out that not only one straight, but two {111} growth fronts are established in a V‐formation, corresponding to the outer {110} and {112} facets, as shown in Figure 7(c). Although this constellation can be regarded as energetically less favorable, it cannot be ruled out in the current investigations. For example, a V‐structure could explain why particles grow along a curved trajectory during a very long pre‐anneal growth, as shown at 120 s in Figure 3(f). Both {111} growth facets could have a different growth rate so that one facet dominates. In addition, other factors such as impurities can lead to a change in the direction of growth.

During heating up to 600 °C and subsequent annealing, the III–V pedestal may be partially reabsorbed by the Au particle. Some particles are nearly absorbed, allowing for free diffusion across the substrate. This mainly affects formation A and particles with a brief pre‐anneal growth procedure, causing them to agglomerate and leave their grid positions. For formations B to D, with longer pre‐anneal growth, the base structure is not fully absorbed, as depicted in Figure 7(d). The {111} growth facet acts as the absorption facet, with regression limited by annealing time. Formation D retains the largest base area due to uniform annealing conditions. In Figure 7(d) the foremost contact point of the Au particle with the substrate remains, so that the Au particle volume has to be distributed over a larger contact area toward the substrate. The Au particle changes shape, becoming longer and flatter while appearing with a smoother surface due to the liquid phase of the particle, with smaller facets, as shown in the SEM images in Figure 6.

Cooling to the NW growth temperature of 430 °C reduces the solubility of Ga in Au, causing GaAs crystallization, shown in Figure 7(e). The liquid phase of the particle determines the growth facet, leading to VLS‐based NW growth in the <111>B direction. This growth occurs over the entire particle‐substrate interface, displacing the particle in the <111>B direction. The process is limited by the Ga concentration in the Au particle and continues until supersaturation ends.

To further explore the flexibility of our approach, we have also extended the study to include other Au disk geometries, which enables the fabrication of NWs with different diameters. Such diameter variations are of particular interest for applications where device performance depends on structural parameters, such as solar cells, solar water splitting, and other (opto‐)electronic devices. Therefore, our ability to customize the geometry of Au particles through UTAM processing and Au disk design enables us to fabricate versatile, application‐oriented NW arrays.

**Figure 8:**
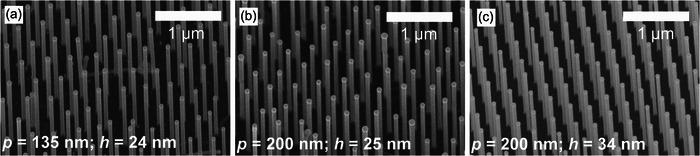
SEM images of NW samples with optimized process parameters for different *p* and *h*. a) *p*=135 and *h*=24 nm. b) *p*=200 and *h*=25 nm. c) *p*=200 and *h*=34 nm. All samples exhibit a value of *a*=400 nm. Images taken under 30° tilt.

By employing the approach of adjusting the pre‐anneal growth time, the optimum time for various volumes of Au particles can be identified, and successful NW growth can be generated in an array arrangement. The regulation of the Au volume involves the adjustment of the UTAM pore size *p* and the initial height *h* of the Au disks, which can be influenced by the PVD time. As the diameter of the Au particle determines the subsequent NW diameter, the thickness of the NWs can be fine‐tuned. Consequently, it is feasible to obtain NWs with varying thicknesses. **Figure** **8** demonstrates vertically grown NWs obtained using UTAM‐structured Au disks with different geometric configurations and thus varying Au particle volumes. This was achieved by adjusting the pore opening *p* to values to 135 and 200 nm, as well as the Au disk height *h* to values of 25 and 34 nm, as measured by atomic force microscopy (AFM) (see Figure S1, Supporting Information). For each Au volume, the pre‐anneal growth times are modified, resulting in NWs with diameters of 79 ± 4 nm (Figure 8(a)), 91 ± 4 nm (b), and 97 ± 4 nm (c) as measured from SEM images. The diameters were determined using SEM images of 20 individual wires. The pre‐anneal growth times corresponded to (b) 65 s, (c) 75 s, and (d) 77 s. This demonstrates that our approach – namely the pre‐anneal growth step – allows the supply time of the precursors to be fine‐tuned according to the specific volume of the Au particle. This ensures effective particle anchoring, and is not restricted to a single configuration. In summary, the pre‐anneal growth procedure is a decisive process for stabilizing the Au particle positions prior to the high‐temperature annealing required for vertical NW growth.

## 3 Conclusion

We employ a refined fabrication routine for vertically aligned III–V semiconductor NWs by combining UTAM pattering with VLS MOVPE growth. A central innovation of this process is the implementation of a pre‐anneal growth procedure, which stabilizes the position of Au catalyst particles prior to high‐temperature annealing – an essential prerequisite for maintaining array fidelity during NW formation.

Our results show that the duration of this pre‐anneal growth step is critical: if too short, Au particles tend to agglomerate into larger clusters and leave their array sites; if too long, excessive planar base growth displaces the particles and degrades the array regularity. Thus, an optimum time window, dependent on the Au particle volume, must be determined for each combination of array geometry parameters (*h* and *p*).

A detailed analysis of pedestal formation revealed that Au particles advance along crystallographically preferred directions, driven by material deposition at the Au–GaAs interface. These findings were used to develop a comprehensive growth model that captures the key physico‐chemical processes occurring during the respective MOVPE steps.

The study further demonstrates that the initial UTAM‐defined parameters, such as Au nanodisk/particle volume, pore size *p*, and height *h*, directly affect the resulting NW geometry. Since NW dimensions govern both optical and electronic properties, this level of control is essential for tailoring NW arrays to specific device application. In summary, our approach provides a reliable and scalable method for fabricating well‐ordered NW arrays with tunable geometry – opening new pathways for large‐scale NW integration in (opto‐)electronics and solar energy devices.

## 4 Experimental Section

### UTAM Fabrication

UTAM, an ultrathin nanoporous AAO, utilizes a nanoimprint‐assisted anodization process for precise nanostructuring for various applications, especially for (photo)electrochemical energy conversion and storage.^[^ ^26^, ^29^, ^46^ ^]^ It facilitates high reproducibility while maintaining unique, customized physical properties,^[^ ^47^, ^48^ ^]^ and enables the efficient fabrication of well‐defined arrays of nanostructures with unique and controllable physical properties.^[^ ^49^, ^50^ ^]^ UTAM masks have uniform pore size and long‐range ordered pores over large areas (typically several square centimeters, up to 4 inches), which can be either rectangular or hexagonal in shape.^[^ ^26^, ^49^ ^]^

The basic idea is to use a mask made of porous Al with a layer thickness of a few hundred nanometers, whose pores can be used for further processing. The pores have the advantage that their diameter can be adjusted in the range of a few hundred nanometers. Figure 1 illustrates the step‐by‐step procedure for fabricating the UTAM and subsequently depositing the Au particles. A detailed overview of the chemicals employed for UTAM fabrication can be found in Table T1 of the supporting information. The starting material for the process is a high‐purity (99.999%) Al foil with a thickness of 200 µm. After cleaning with acetone, the foil is electrochemically polished in a solution of 1 part perchloric acid (HClO_4_) and 7 parts ethanol (C_2_H_6_O), at a potential of 30 V and 0 °C, with constant stirring for 2 to 3 min.^[^ ^28^, ^32^ ^]^ As reported previously,^[^ ^51^ ^]^ a nickel stamp is pressed onto the sample for three minutes using an oil press under a hydraulic pressure of ≈14.0 MPa, as illustrated in Figure 1(a).^[^ ^52^ ^]^ After removing the stamp, the highly ordered impressions appear as a negative of the stamp's thorns on the foil. In a controlled anodizing step in (b), an oxide layer is produced that uses the imprints as nuclei and develops pores from them. The anodization process is carried out at a constant voltage of 160 V in a 0.4 m phosphoric acid solution at a temperature of 8 °C for a period of 5 min. Following anodization, the mask is first affixed to poly(methyl methacrylate) (PMMA) and dried at 40 °C for 1 h. This makes it durable enough to be used as a UTAM substrate, enhancing its stability.^[^ ^51^ ^]^ The Al foil on the back is then removed using a solution of copper chloride (CuCl_2_, 85 wt.%) and hydrochloric acid (HCl, 15 wt.%). Following wet‐chemical etching in a phosphoric acid bath (H_3_PO_4_, 5 wt.%), the barrier layer is removed, which opens and expands the pores. Subsequently, the PMMA layer is removed using acetone, and the UTAM mask is thoroughly rinsed with distilled water and then dried. To transfer the UTAM mask onto the III–V substrate, the substrate is placed in distilled water, allowing the mask to be carefully aligned and attached to the sample. Once the transfer is complete, as depicted in Figure 1(c), the substrate with the applied UTAM mask is removed from the water and dried. As shown in Figure 1(d), with PVD an Au layer of any desired thickness can be deposited to fill the pores of the UTAM at a deposition rate of ≈1–2 nm min^−1^. During this step, the sample is rotated at ≈20 revolutions per minute to form a homogeneous layer. Subsequently, the samples undergo an annealing‐like process during the 30 min de‐vacuuming process, as the temperature decreases from the deposition temperature (≈200 °C) to room temperature. Finally, the mask can be removed from the substrate using a nitrogen gun, as shown in Figure 1(e). What remains are the highly ordered Au particles, which have adopted the parameters of the UTAM, such as diameter, spacing, and thus also order.

### MOVPE Sample Preparation

For the reported investigation, GaAs pedestals or NW structures were prepared by MOVPE in a horizontal low‐pressure Aixtron AIX 200 MOVPE reactor with H_2_ as carrier gas at 50 mbar. In all cases, p‐doped GaAs (111) type B substrates were used.

At the beginning of the MOVPE process, the Au particle is in a solid state with a disk‐shaped structure, as defined by the UTAM mask, depicted in Figure 1(g). Tertiarybutylarsine (TBAs) is introduced as a precursor with a molar flow rate of 4.9 × 10^−5 ^mol min^−1^ to ensure an arsenic overpressure when T > 300 °C. Introducing a longer pre‐anneal growth step of up to 120 s (analogous to the short pre‐anneal nucleation step of 60 s in ref. [25]), during which the III‐component precursor trimethylgallium (TMGa) is introduced into the MOVPE reactor, initially enriches the Au disk and forms a Ga‐Au alloy. The molar flow rate of the Ga precursor is 1.9 × 10^−5^ mol min^−1^. It should be noted that only a small proportion of the group V component is soluble in Au. Supersaturation results in the growth of a small NW pedestal, as depicted in Figure 1(g), analogous to conventional NW growth, but at lower temperatures. For pre‐anneal growth, a temperature of 320 °C was chosen, where it can be assumed that the Au particle is in the solid phase, forming during VSS growth that fixes the Au particles in place. Due to the interplay between providing a sufficiently large time window for the precise adjustment of the pre‐anneal growth time to the given volume of Au particles, as discussed for Figure 3, and ensuring reduced defect densities in the pedestal, the selected temperature must be chosen carefully to find the right compromise. If the temperature is set too high, complete pyrolysis of the precursor occurs (400 °C for TBAs and 525 °C for TMGa),^[^ ^53^, ^54^ ^]^ leading to rapid pedestal growth. As a result, the parameter window for particles to become sufficiently anchored and for undesirable planar pedestal growth to be prevented becomes significantly smaller. Conversely, if the temperature is too low, the pyrolysis becomes insufficient to provide enough group III and group V components. Also, surface mobility is reduced, which can promote the formation of defects in the pedestal.

After the pre‐anneal growth step, the supply of TMGa was stopped and the temperature was increased to 600 °C. During this annealing step, a molar flow rate of 9.4 × 10^−5 ^mol min^−1^ of TBAs was maintained in order to create an arsenic overpressure and thereby suppress arsenic desorption from the substrate surface. At 600 °C, the III–V base material is partially reabsorbed by the Au particle as depicted in Figure 1(h). This leads to anchoring on the substrate surface and ensures that the particles remain in place and are available for the subsequent NW growth (see Figure 1(i)). The heating and cooling times between 320 and 600 °C are system‐dependent and are typically range 5 min. NW growth was initiated at 430 °C by supplying TMGa at a molar flow rate of 1.9 × 10^−5 ^mol min^−1^ together with a TBAs molar flow rate of 4.9 × 10^−5 ^mol min^−1^. Both precursors were introduced for 5 min. The growth process was terminated by interrupting the TMGa molar flow, cooling the sample to room temperature. The TBA flow was continued during cooling and was stopped below 300 °C to complete the process and prepare the sample for further analysis. SEM images were taken before and after the MOVPE process using a HITACHI S4800 SEM.

## Conflict of Interest

The authors declare no conflict of interest.

## Supporting information



Supporting Information

## Data Availability

The data that support the findings of this study are available from the corresponding author upon reasonable request.
