# Supplementary material for: Pattern Fidelity of Vertically Aligned GaAs Nanowire Arrays
Source: Small. 2025 Oct 14;21(47):e06173. doi: 10.1002/smll.202506173 (PMC12658925; doi:10.1002/smll.202506173)
Supplement: Supplementary file 1 — Supporting Information [file SMLL-21-e06173-s001.pdf]

NANO · MICRO
small

Supporting Information

for *Small*, DOI 10.1002/smll.202506173

Pattern Fidelity of Vertically Aligned GaAs Nanowire Arrays

*Juliane Koch, Jiajia Qiu, Chris Yannic Bohlemann, David Ostheimer, Huaping Zhao, Peter Kleinschmidt, Yong Lei and Thomas Hannappel**

Supporting Information

Pattern Fidelity of Vertically Aligned GaAs Nanowire Arrays

*Juliane Koch, Jiajia Qiu, Chris Yannic Bohlemann, David Ostheimer, Huaping Zhao, Peter Kleinschmidt, Yong Lei and Thomas Hannappel**

J. Koch, C. Bohlemann, D. Ostheimer, P. Kleinschmidt, T. Hannappel
Fundamentals of Energy Materials, Institute of Physics & IMN MacroNano, Technische Universität Ilmenau, 98693 Ilmenau, Germany

J. Qiu, H. Zhao, Y. Lei
Fachgebiet Angewandte Nanophysik, Institut für Physik & IMN MacroNano, Technische Universität Ilmenau, 98693 Ilmenau, Germany

E-mail: Thomas.Hannappel@tu-ilmenau.de

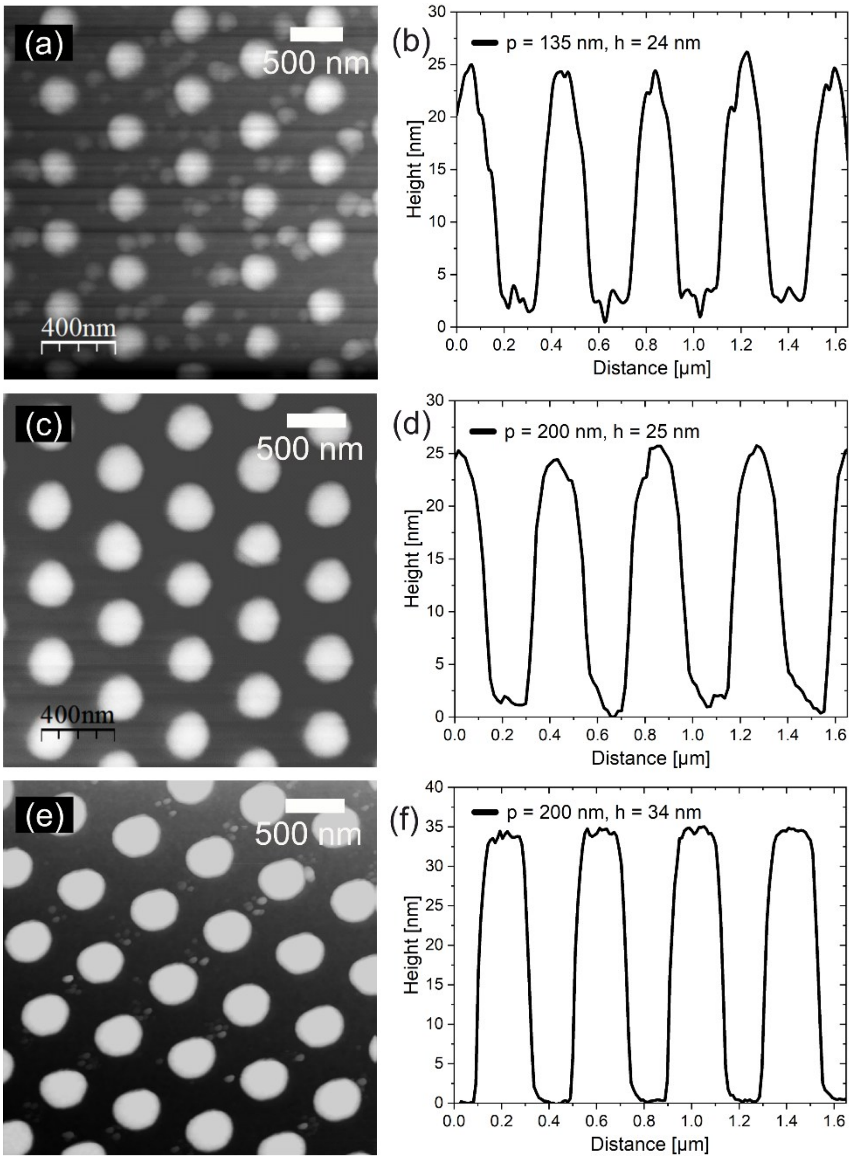

Figure S1. AFM measurements of used samples. (a), (c) and (e) AFM images, and (b), (d) and (f) line profiles. (a) and (b) sample with p = 135 nm, h = 24 nm. (c) and (d) sample with p = 200 nm, h = 25 nm. (e) and (f) sample with p = 200 nm, h = 34 nm.

Figure S1 depicts AFM measurements of three samples, which are used for NW growth, shown in Fig. 8. The height of the Au disks can be estimated to (a) 24 nm, (c) 25 nm and (e) 34 nm. The AFM measurements are performed by a Bruker AFM Dimensions ICON XR and analyzed with NanoScope Analysis 3.00 software.

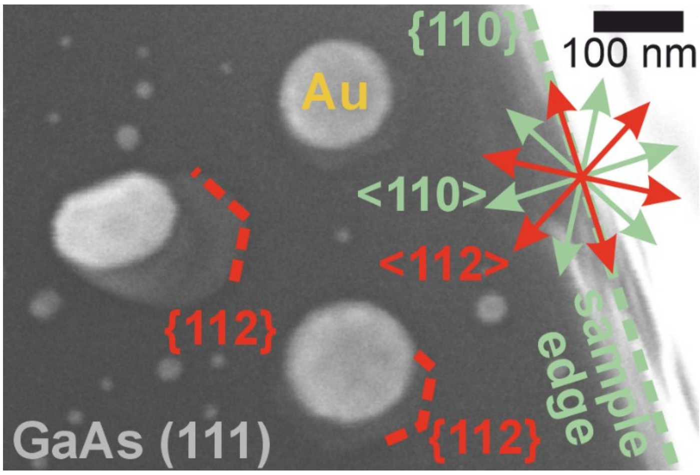

Figure S2. Top-view SEM image of a GaAs(111)B substrate with marked orientations and planes.

Figure S2 shows an SEM image of the GaAs(111)B substrate in the top-view. The sample edge is visible in the {110} direction on the right-hand side, with the GaAs pedestals and {112} planes visible on the left-hand side. Brighter circles indicate the Au particles.

Name	*Chemical purity*	*Supplier sources*
$NiSO_4$	100%	VWR® Chemicals
$NiCl_2$	98%	VWR® Chemicals
H_3BO_3	100%	VWR® Chemicals
$CuCl_2$	99.1%	VWR® Chemicals
$HClO_4$	61%	VWR® Chemicals
C_2H_6O	96.3%(v/v)	VWR® Chemicals
H_3PO_4	85.5%	VWR® Chemicals
PMMA	-	MICRO CHEM
CH_3COCH_3	-	Sigma-Aldrich®
HCl	35-39%	VWR® Chemicals

Table T1. Overview of chemicals used for UTAM fabrication.

Table T1 presents all relevant chemicals used in the UTAM fabrication process, specifying their respective purity and supplier.
